# Prerequisites for the clinical implementation of a markerless SGRT-only workflow for the treatment of breast cancer patients

**DOI:** 10.1007/s00066-022-01966-7

**Published:** 2022-07-04

**Authors:** Tim-Oliver Sauer, Oliver J. Ott, Godehard Lahmer, Rainer Fietkau, Christoph Bert

**Affiliations:** 1 Department of Radiation Oncology, Universitätsklinikum Erlangen, Friedrich-Alexander-Universität Erlangen-Nürnberg, Universitätsstr. 27, 91054 Erlangen, Germany; 2 Comprehensive Cancer Center Erlangen-EMN, Erlangen, Germany

**Keywords:** Surface-guided radiation therapy, Markerless radiation therapy, Breast neoplasms, Interfraction motion management, Intrafraction motion management

## Abstract

**Purpose:**

A markerless workflow for the treatment of breast cancer patients has been introduced and evaluated retrospectively. It includes surface-guided radiation therapy (SGRT)-only positioning for patients with small cone beam CT (CBCT) position corrections during the first five fractions. Prerequisites and the frequency of its clinical application were evaluated, as well as potential benefits in terms of treatment time and dose savings, the frequency of CBCT scans, and the accuracy of the positioning.

**Methods:**

A group of 100 patients treated with the new workflow on two Versa HD linacs has been compared to a matched control group of patients treated with the former workflow, which included prepositioning with skin markings and lasers, SGRT and daily CBCT. The comparison was based on the evaluation of logfiles.

**Results:**

Of the patients treated with the new workflow, 40% did not receive daily CBCT scans. This resulted in mean time savings of 97 s, 166 s and 239 s per fraction for the new workflow, for patients treated without daily CBCT and for SGRT-only fractions, respectively, when compared to the old workflow. Dose savings amounted to a weighted computed tomography dose index reduction of CTDI_W_ = 2.56 cGy on average for normofractionated treatment and weekly CBCTs, while for patients not treated with daily CBCT, SGRT-based positioning accuracy was 5.2 mm for the mean translational magnitude, as evaluated by CBCT.

**Conclusion:**

For 40% of the patients, after five fractions with small CBCT corrections, the workflow could be changed to SGRT-only positioning with weekly CBCT. This leads to imaging dose and time savings and thus also reduced intrafraction motion, potentially increased patient throughput and patient comfort, while assuring appropriate positioning accuracy.

## Introduction

Precise patient positioning during radiation therapy is pivotal for correct application of the desired dose distribution. Generally, a combination of prepositioning using skin markings and lasers and an image guidance-based correction is used for a wide range of entities. This approach comes with the drawbacks of low prepositioning accuracy, patient disturbance due to the skin markings and their regular renewal, and the exposition to additional ionizing imaging dose. Furthermore, continuous control of the patient’s position during treatment is not possible.

At the same time, surface-guided radiation therapy (SGRT) has been established as a powerful alternative for patient positioning and control [1, 2]. Through the detection of the full patient surface, superior information about the patient’s position is obtained compared to methods using pointwise information like skin markings. SGRT can therefore be used as a tool for prepositioning the patient with higher accuracy than with lasers and render the use of skin markings redundant [3–5]. On the other hand, it has been shown that under certain conditions, SGRT is potentially able to replace even the IGRT-based position correction [4, 6–9] and decrease treatment time and imaging dose [10]. Protocols with only weekly imaging verification (meaning one cone beam CT (CBCT) every five treatment fractions) have been proposed [8] and reported in the literature [11, 12]. For such an approach, it must be clear under which conditions the positioning accuracy using SGRT-only is maintained.

In this study, we evaluated the introduction of a markerless clinical workflow for the treatment of breast cancer patients. No skin markings were used and prepositioning was done only with SGRT. Based on an analysis of the CBCT-correction shifts that followed prepositioning during the first five treatment fractions, an individual positioning scheme was chosen for every patient—either the patient was treated with a daily CBCT-position correction, or with SGRT-based positioning only and CBCT correction on a weekly basis for regular control. Prerequisites and the frequency of the clinical application of the new workflow were evaluated and the positioning accuracy, time and dose savings, compared to the former workflow with skin markings and daily CBCT, were analyzed.

## Materials and methods

### Patient treatment workflow

The analyzed data were obtained from treatments carried out as part of the clinical routine at the Department of Radiation Oncology, Universitätsklinikum Erlangen, on two Versa HD linacs, equipped with the Hexapod treatment couch, the XVI CBCT system (all Elekta, Stockholm, Sweden) and the surface scanner AlignRT (Version 5.1.2; VisionRT, London, UK).

The previous (“old”) workflow involved prepositioning with skin markings (drawn with a permanent pen) and lasers, further alignment using the surface scanner, followed by a 6-degrees of freedom (DOF) CBCT-based position correction on a daily basis. The weighted computed tomography dose index of a CBCT acquired with the XVI system with clinical parameters (100 kV, 10 mA, 10 ms, 220 projections) was approximately CTDI_W_ = 1.5 mGy. The use of skin markings involved the necessity of renewal after treatment on an irregular basis.

**Fig. 1:**
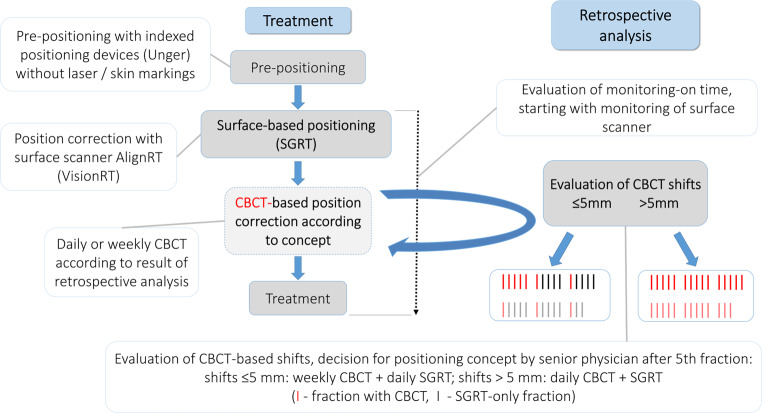
New workflow without laser-based prepositioning and individually chosen positioning concept. *CBCT* cone beam computed tomography, *SGRT* surface-guided radiation therapy

The newly introduced (“new”) workflow did not involve skin markings (Fig. 1). A rough prepositioning of the patient was carried out by indexing the positioning devices (UNGER Medizintechnik, Mülheim-Kärlich, Germany). In this way, the target volume was brought close enough to isocenter that it entered the field of view of the surface scanner (approximately within 10 cm from isocenter). From there, the positioning was done with the SGRT system, minimizing SGRT-based shifts if they exceeded the clinical tolerances (3 mm and 3°, respectively). Rotational deviations were corrected manually, sometimes including an adjustment to the patient’s posture with the positioning devices, since this was not possible with the subsequent CBCT. During the first five fractions, daily CBCTs were acquired and registered to the planning CT. For that, RTTs used a rigid grey-value-based registration algorithm and corrected manually if necessary. Since this procedure was used for testing the SGRT-only situation, it had to be done in 3‑DOF because rotational movements of the treatment couch could not be performed based on SGRT (limited functionality of interface). The CBCTs and the applied shifts of the first five fractions were evaluated individually for every patient by senior physicians. A positioning scheme for the rest of the treatment was chosen based on clinical experience and the size of the shifts. A value of 5 mm for the translational magnitude served as a point of orientation for the threshold, complemented by a qualitative evaluation of the registration involving rotations. Either the patient was positioned every day like the first five fractions, i.e., including daily CBCTs, or only once every five fractions with CBCT and for the rest of the fractions with SGRT-only. Changes from one scheme to the other during treatment based on consecutive CBCT evaluation were allowed for, but did not occur. After a CBCT-based position correction, a new surface reference capture was always acquired for both workflows.

### Data analysis

For each workflow, we retrospectively identified a group of 100 patients with comparable properties with regard to target volume (with or without lymph nodes), treatment scheme (normo- or hypofractionated), and breathing technique (free breathing or deep inspiration breath hold [DIBH]; Table 1).

**Table 1:** Characteristics of patient base of old (P_OLD_) and new workflow (P_NEW_)

	CBCT	Period of treatment	Patients/fractions	Hypofractionated 15 × 2.67 Gy	Normofractionated 28 × 1.8 Gy	DIBH	Free breathing	Without lymph nodes	With lymph nodes
P_NEW_	According to concept	01/2020–11/2020	100/2186	43	57	20	80	49	51
P_OLD_	Daily	01/2019–01/2020	100/2151	38	62	20	80	49	51

*DIBH* deep inspiration breath hold, *CBCT* cone beam computed tomography

The entire treatment time, meaning the time between the patient’s entrance and exit of the treatment room, was not directly accessible from our data. In order to compare different workflows, we used the logfiles of the SGRT system and at some points additionally those of the treatment couch. From the former, we extracted the time that the surface scanner was monitoring the patient’s surface, starting with the moment of switching on the monitoring and ending when the patient went out of sight of the scanner at the end of treatment (“monitoring-on time”). Average monitoring-on times were calculated and stratified according to the positioning scheme. Based on experience and plausibility, fractions with monitoring-on times lower than 150 s or higher than 2000 s were excluded. For these fractions (18 of 2204 and 24 of 2175 fractions for the new and old workflow, respectively), logfiles were not recorded properly, as was evaluated individually. Monitoring-on times were analyzed with respect to potential trends along the treatment (i.e., fraction number) and starting date of the treatment in order to detect potential learning effects. The treatment couch logfiles contain a great amount of information, but data was rarely complete and only available for treatments in 2020, i.e., patients treated with the new workflow. Information about timing of the call of patients in the patient information management system (Mosaiq, Elekta), start and end of CBCT, and treatment fields could be evaluated. Where corresponding data were available, SGRT and treatment couch logfiles were compared and examined for correlation. Furthermore, the clinically applied CBCTs of different patient subgroups were evaluated statistically with respect to average value and their frequency distribution.

The two-sample Student t‑test for unequal variances (Welch’s t‑test) was applied to the data in order to check for statistical significance. Results were rated as not significant (*p* ≥ 0.05) or significant (*p* < 0.05). All calculations and plotting were performed with Anaconda 3.1/Python 3.4.

## Results

### CBCT frequency and magnitude of shifts

Of 100 patients treated with the new workflow (P_NEW_), 60 patients received daily CBCT (P_NEW,D_). Of the 40 patients that did not receive daily CBCTs (P_NEW,W_), 28 patients received weekly CBCTs; 12 patients received CBCTs more often than weekly, but not daily. The characteristics of the overall patient collective with respect to, for example, treatment technique (Table 1) was conserved within the subgroups of patients receiving daily or less than daily CBCT, respectively. On average, patients from group P_NEW,W_ received 0.43 CBCTs per fraction (0.39 and 0.54 per fraction for normo- and hypofractionated treatment, respectively), whereas group P_NEW,D_ received 0.99 CBCTs per fraction. Overall, patients treated with the new workflow P_NEW_ received 0.76 CBCTs, and patients treated with the old workflow P_OLD_ received 0.98 CBCTs per fraction on average (for remaining fractions, orthogonal kV imaging was used). Based on these data, the omission of CBCTs led to the following average dose savings for patients treated without daily CBCT: for normofractionated treatment, on average 60% of the CBCTs (i.e., 16.8 CBCTs) and thus of the dose (i.e., CTDI_W_ = 2.52 cGy) were omitted. Due to the reduced number of fractions, this proportion was lower for hypofractionated treatment, namely 45% of the CBCTs (i.e., 6.75 CBCTs) and thus of the dose (i.e., CTDI_W_ = 1.01 cGy) were omitted on average.

**Fig. 2:**
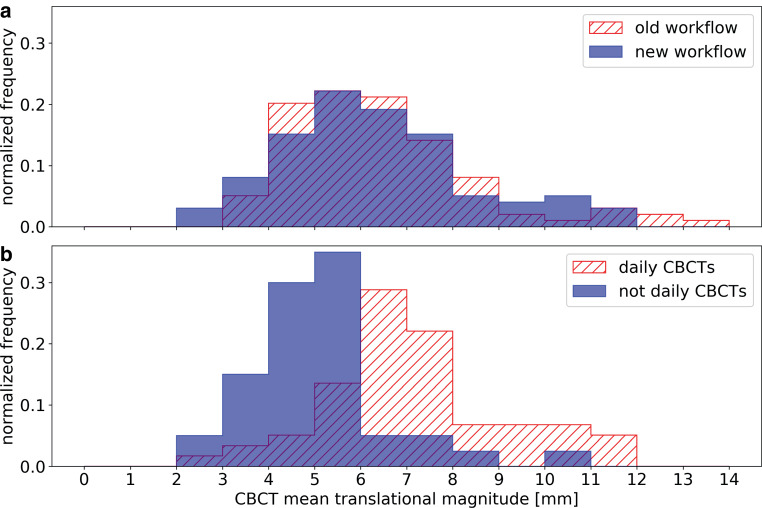
Normalized frequency distribution of the mean translational magnitude of clinically applied cone beam computed tomography (CBCT) shifts after surface-guided radiation therapy (SGRT)-based alignment; comparison of patients treated with the old and new workflow (**a**), and of patients treated with the new workflow receiving CBCTs on a daily and less than daily basis (**b**), respectively

The average of the translational magnitude of the clinically applied CBCT shifts was 6.6 mm for the old and 6.9 mm for the new workflow (*p *$\ll$0.05). For the subgroups based on the applied positioning scheme, we obtained values of 7.5 mm and 5.2 mm for P_NEW,D_ and P_NEW,W_, respectively (*p *$\ll$0.05). The frequency distribution showed also big differences for both subgroups (Fig. 2b): 85% of the patients of P_NEW,W_ and 23.7% of the patients of P_NEW,D_ had CBCT shift magnitudes smaller than 6 mm. Old and new workflow showed comparable histograms (Fig. 2a).

### Treatment time

**Fig. 3:**
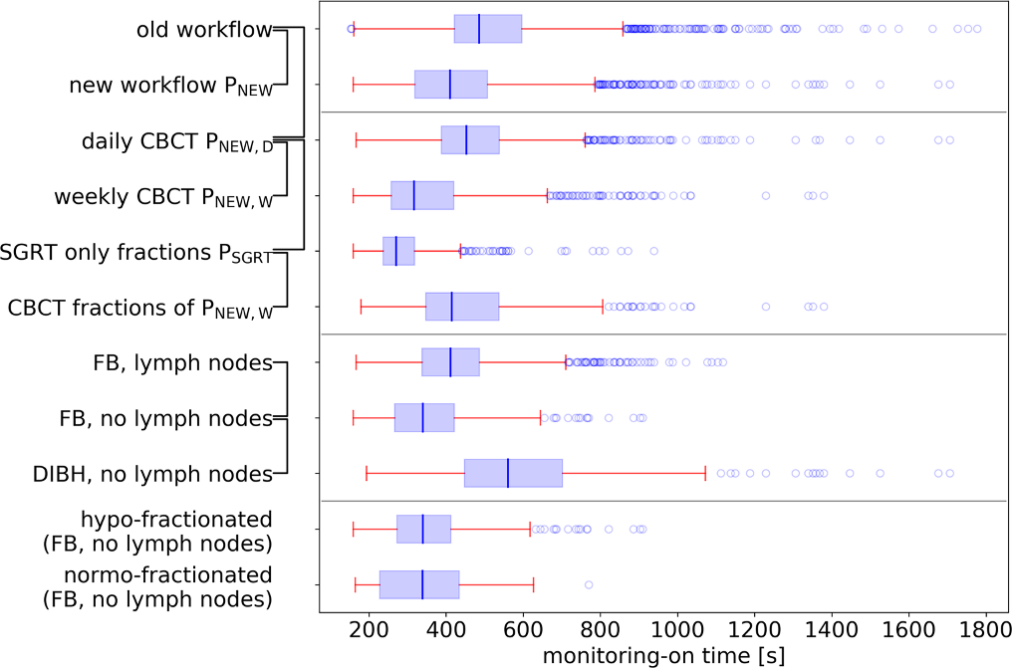
Boxplot of monitoring-on times of different subgroups of patients and fractions (*boxes* extend from first to third quartile, *whiskers* 1.5 interquartile range from boxes; fliers marked by *circles*), with *brackets* indicating statistically significant differences between the respective times. *FB* free breathing, *DIBH* deep inspiration breath hold, CBCT cone beam computed tomography, *SGRT* surface-guided radiation therapy

We found significant time savings for the new workflow compared to the old workflow (t_OLD_ = 533 s, Table 2 and Fig. 3). For all patients treated with the new workflow, monitoring-on time was reduced by 97 s on average (t_NEW_ = 436 s, *p *$\ll$0.05). For patients treated without daily CBCT, the reduction amounted to 166 s (t_NEW,W_ = 367 s, *p *$\ll$0.05), and the largest reduction of 239 s was observed for fractions without CBCT (t_SGRT_ = 294 s, *p *$\ll$0.05). The difference between monitoring-on times of CBCT fractions of P_NEW,D_ (484 s) and P_NEW,W_ (470 s) was not significant. Results for patient subgroups, sorted according to type of treatment, showed significant differences for lymph node irradiation vs. no lymph node irradiation and free breathing vs. DIBH treatment, but not for normo- vs. hypofractionated treatment.

**Table 2:** Mean monitoring-on times of different subgroups of patients and fractions

		Patients (*n*)	Fractions (*n*)	Monitoring-on time (s)	*p*-value
A	Old workflow (P_OLD_)	100	2151	533	–
B	New workflow (P_NEW_)	100	2186	436	A–B: $\ll$0.05
C	Daily CBCT (P_NEW,D_)	60	1293	484	A–C: $\ll$0.05
D	Less than daily CBCT (P_NEW,W_)	40	893	367	C–D: $\ll$0.05
E	SGRT-only fractions of P_NEW,D_ (P_SGRT_)	40	521	294	C–E: $\ll$0.05
F	CBCT fractions of P_NEW,W_	40	388	470	C–F: 0.19 E–F: $\ll$0.05
G	FB, with lymph nodes	51	1388	425	–
H	FB, without lymph nodes	29	480	359	G–H: $\ll$0.05
I	DIBH, without lymph nodes	20	318	601	H–I: $\ll$0.05
J	Hypofractionated, FB, without lymph nodes	24	347	363	–
K	Normofractionated, FB, without lymph nodes	5	133	348	J–K: 0.26

*FB* free breathing, *DIBH* deep inspiration breath hold, *CBCT* cone beam computed tomography, *SGRT* surface-guided radiation therapy

**Fig. 4:**
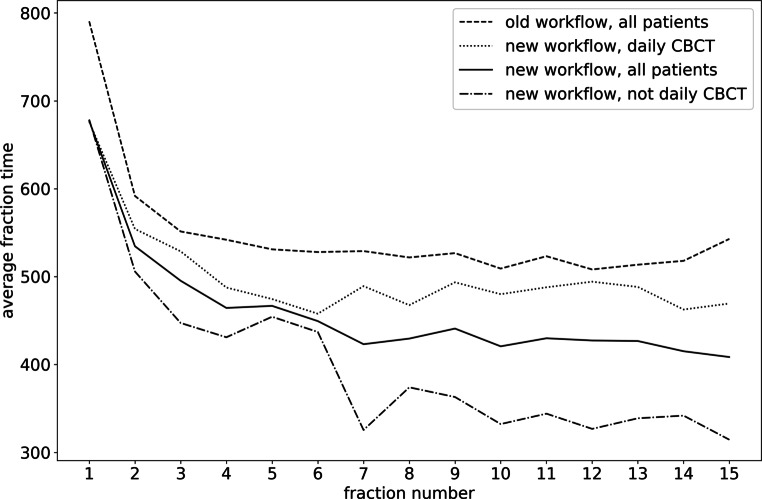
Fraction-wise averaged monitoring-on time for patients treated with the new workflow, with and without daily cone beam computed tomography (CBCT), respectively, limited to the first 15 treatment fractions in order to have comparable datasets

Monitoring-on times showed a decrease along the treatment, i.e., with increasing fraction number (Fig. 4). For patients treated with the old workflow or the new workflow with daily CBCT, average time of the first fraction (790 s and 676 s, respectively) was 48% and 40% higher than the overall average, respectively. No such development was apparent for the patient-wise averaged monitoring-on time with respect to the starting date of treatment.

From fractions of the new workflow with CBCT acquisition and available treatment couch data (581 fractions), we obtained an average time of 159 s for the acquisition of a CBCT, including registration and corresponding couch shift. Similarly, we obtained an average of 218 s for radiation time, i.e., from first beam-on to last beam-off (data of 2218 fractions). We found a high correlation between SGRT monitoring-on time and data obtained from couch logfiles for times starting either with the call of the patient in the patient management system (t_HEX,P_, Pearson r = 0.76, data of 1988 fractions) or the start of CBCT acquisition (t_HEX,C_, r = 0.81, data of 558 fractions), each stopping with the end of radiation. The corresponding mean time differences between times of couch and surface scanner (t_ART_) were t_HEX,P_ − t_ART_ = 129 s and t_HEX,C_ − t_ART_ = −59 s, respectively.

## Discussion

The CBCT shifts were significantly higher for patients receiving daily CBCT (P_NEW,D_) than for those not receiving daily CBCTs (P_NEW,W_), as is to be expected since the individual positioning concept is chosen on the basis of these shifts. On the other hand, in spite of overall differing shift frequency distributions for the two subgroups (Fig. 2b), some patients with low shift magnitude were still treated with daily CBCT, and some with higher shift magnitude (above threshold value of 5 mm) with fewer CBCTs. This is because senior physicians also used their clinical experience and considered other observations for their decision, including positioning inaccuracies that persist after the 3‑DOF CBCT position correction, which potentially could be reduced by using 6‑DOF position correction (which technically is only possible in combination with CBCT, but not with SGRT). The mean CBCT shift of the old workflow was slightly, but significantly lower than that of the new workflow. This may be due the lack of CBCT-based rotational position corrections, which can lead to higher translational shifts. For 40% of patients, the accuracy of the SGRT-only positioning could be kept within a clinically acceptable level (average translational magnitude of 5.2 mm). Following the reasoning of Wiant et al., the time savings counterbalance potential loss of positioning precision because of reduced intrafraction motion [13]. In a study of breast cancer patients with lymph node irradiation treated with tomotherapy, Crop et al. reported on intrafractional shift magnitudes of 3.8 mm after 10 min [8]. The fact that SGRT-only leads to time savings, reduced intrafractional motion can be expected, which further emphasizes the accuracy of the method.

Monitoring-on times of patients treated with daily CBCTs (P_NEW,D_) were 117 s longer on average than for those without daily CBCT (P_NEW,W_). This difference does not stem from the different patient collective (i.e., the treatment of patients with smaller CBCT shifts might be easier and therefore faster), underlined by comparable average times of fractions with CBCT for either of the positioning concepts (470 s and 484 s, *p* = 0.19, Table 2). For fractions of P_NEW,W_ without CBCT acquisition, the monitoring-on time was 176 s lower than for those with CBCT. This is in good accordance with the time needed for the acquisition of a CBCT of 159 s, estimated from the treatment couch logfiles. It is not completely clear where the average difference of 49 s between monitoring-on times of the old workflow and the daily-CBCT group of the new workflow stems from exactly. It is possible that it is partly the result of the additional laser-based prepositioning and the related periodic renewal of skin markings after treatment, which was not used in the new workflow. Generally, these times do not overlap with the monitoring-on times of the surface scanner though.

The average difference between the monitoring-on time and the Hexapod time starting with CBCT acquisition of 59 s may be viewed as the time needed for positioning the patient with the surface scanner prior to CBCT acquisition. The difference between monitoring-on time of SGRT-only fractions (294 s) and radiation time (218 s), i.e., 76 s, is thus the time for surface-based positioning of the patient and preradiation preparation like gantry movement to the start position and RTTs leaving the treatment room. Based on the comparison between surface and couch data, the time between the call of the patient and the beginning of surface-based monitoring was 129 s. We also found some kind of learning effect along the treatment, apparent in the decrease of monitoring-on time. DIBH treatments were significantly longer (242 s) than free breathing because of time consuming breath hold control; treatment with and without lymph node irradiation differed by 66 s on average because of a longer radiation time (more treatment fields).

The clinical protocol that was used for the CBCT acquisition in both workflows comprises strongly dose-optimized parameters. The low number of projections (220) and low tube current of 0.1 mAs per frame are on the lower bounds of previously assessed parameters [14], resulting in a dose of CTDI_W_ = 1.5 mGy, while maintaining sufficient image quality. The omission of CBCT acquisitions within the new workflow still led to significant imaging dose savings. On average, 60% and 45% of the dose could be saved with weekly CBCT acquisition for normo- and hypofractionated treatment, respectively. The relatively higher dose saving for normofractionated treatment is a result of the proportionally longer treatment after the initial evaluation phase. The dose savings due to CBCT omission were not as big as the reduction due to optimized CBCT parameters when compared to the manufacturer’s standard chest protocol (CTDI_W_ = 18.3 mGy per CBCT [14]), i.e., nearly 92%. Although the doses are low compared to the therapeutic dose, the avoidance of widespread low intensity dose, especially to the lung and breasts, is still desirable in order to minimize stochastic radiation effects.

The validity of our approach of not having identical patient collectives for old and new workflow with respect to the fractionation scheme was justified by the fact that there was no significant difference between times of normo- and hypofractionated treatment (Table 2 or Fig. 3). Furthermore, the high correlation and agreement between data from the SGRT and the treatment couch system may be taken as evidence of the validity of the used methods. A limitation of this study is the retrospective analysis of both workflows.

The only analysis of the effects of the omission of skin marking that we have encountered in the literature is a study by Jimenez et al. [5]. The authors compared two groups of 10 patients each, receiving accelerated partial breast irradiation (ABPI). For one group, surface-based positioning followed by orthogonal x‑ray-based position correction was used; for the other group, this was preceded by skin-marking/laser-based prepositioning. The treatment times (410 s and 482 s, respectively) cannot be compared directly to the times obtained in the current study because of the different methods of image guidance (kV vs. CBCT) and target volume, fraction dose, etc. Nonetheless, the difference between both treatment times of 72 s corresponds to the omission of laser-based prepositioning, information that we could not evaluate with our data. The kV imaging shift magnitudes after surface imaging were of the order of 5 mm, in good agreement with our results from the CBCT shifts. Our CBCT shift results were also comparable to those of other studies that tested accuracy of surface-based positioning with orthogonal kV [7, 15], MV [4, 12] or CBCT imaging [3]. Pazos et al. showed that initial surface-based setup time was not increased compared to laser-based positioning, and that the number of verification imaging was not increased [11] (Shah reported the same [12]); imaging-based position correction, however, was only performed if shifts reached a threshold level. The authors argue not having found an answer to the question of whether SGRT is potentially capable of reducing the frequency of image guidance. This question was approved by other authors in terms of positioning accuracy [4, 6–8, 13, 16]. Haraldson et al. [10] analyzed patients with different entities, including thorax, treated with tomotherapy. They found that surface guidance could decrease the number of MVCTs significantly (34 to 9), resulting in a 60 cGy dose saving (considerably higher than our results because of mega voltage CTs) and an average time saving of 240 s for thoracic entities. They also reported, however, on large setup deviations for thoracic radiotherapy and were not sure whether it is suitable for surface scanning.

Our study bridges the gaps, showing that there is a sizable number of patients who can be treated by omitting laser-based positioning and daily image guidance and still maintain suitable accuracy by choosing an individual positioning scheme after the first five treatment fractions, which leads to time and dose savings.

## Conclusion

The introduction of a new markerless surface-guided radiation therapy (SGRT)-only workflow, including an individual positioning concept with or without daily cone beam CT (CBCT) acquisition based on the CBCT shifts of the first five fractions, was evaluated. Forty percent of the patients were found suitable for SGRT-only treatment, with image guidance-based verification on a usually weekly basis and positioning accuracy of 5.2 mm translational magnitude on average. Improvements included imaging dose savings of CTDI_W_ = 2.52 cGy, corresponding to a 60% reduction, and CTDI_W_ = 1.01 cGy, corresponding to a 45% reduction, for normo- and hypofractionated treatment, respectively. We found considerable time savings as well, i.e., monitoring-on times per fraction were 97 s shorter on average for the new workflow, 166 s shorter for patients without daily CBCT, and 239 s shorter for SGRT-only fractions, when compared to the old standard workflow. This leads to reduced intrafraction motion, potentially increased patient throughput, reduced nontherapeutic dose and, in combination with the omission of skin markings, higher patient comfort while assuring appropriate positioning accuracy.
